# *Helicobacter pylori* Vaccine: Mechanism of Pathogenesis, Immune Evasion and Analysis of Vaccine Types

**DOI:** 10.3390/vaccines13050526

**Published:** 2025-05-15

**Authors:** Jingwen Gong, Qing Wang, Xing Chen, Junhui Lu

**Affiliations:** 1 The First Clinical Medical College, Shanxi Medical University, Taiyuan 030001, China; gjw258025@163.com (J.G.); was980118@163.com (Q.W.); lujunhui@sxmu.edu.cn (J.L.); 2 Department of Gastroenterology, The First Hospital of Shanxi Medical University, Taiyuan 030001, China

**Keywords:** *Helicobacter pylori*, *Helicobacter pylori* vaccine, pathogenic mechanism of *Helicobacter pylori*, *Helicobacter pylori* immune evasion

## Abstract

*Helicobacter pylori (H. pylori)* is a gram-negative, spiral-shaped bacterium that colonizes the human gastric mucosa, leading to various gastric diseases. *H. pylori* infection has become a pressing public health issue that affects more than 50% of the human population worldwide, almost 40 years after its discovery. Traditional treatments, based on the use of bismuth-based triple and quadruple therapies, are effective while facing a series of problems, such as difficulty in patient compliance, the rise of antibiotic resistance, and possible recurrence of infection. Therefore, the development of an efficacious vaccine against *H. pylori* would be extremely urgent. This review mainly elaborates on the pathogenic mechanism and immune evasion mechanism of *H. pylori*, as well as various strategies adopted in vaccine development, including whole-cell vaccines, subunit vaccines, DNA vaccines, and live vector vaccines. Animal studies and clinical trials demonstrate that *H. pylori* vaccines significantly reduce bacterial load and provide cellular immunity over some time. Multiple studies have clarified the advantages and limitations of each candidate vaccine. Although the development of *H. pylori* vaccines provides benefits to reduce the global burden, there are still significant challenges to developing vaccines in safety, efficacy, and availability. Overcoming these challenges, along with the advancement of vaccine technology, can better prevent and treat *H. pylori* infection.

## 1. Introduction

*Helicobacter pylori* (*H. pylori*), a type of Gram-negative spiral bacterium found on the gastric mucosa, can cause a series of gastric diseases, such as gastric cancer, peptic ulcer, atrophic gastritis, and gastric mucosa-associated lymphoid tissue (MALT) lymphomas [1,2,3,4]. *H. pylori* not only is a significant contributor to chronic gastritis and peptic ulcers, but also causes poor prognosis for diseases outside the digestive system, including the proliferation and invasion of prostate cancer cells [5], eye disorders, metabolism-related conditions, and neurological disorders [6]. Therefore, it’s extremely important for human health to eradicate *H. pylori*. Worldwide prevalence among adults was 52.6% before 1990, although the infection rate of *H. pylori* infection dropped to 43.9% from 2015 to 2022 which was due to the development of screening and treatment technologies. According to a large-scale randomized trial, eradicating *H. pylori* can reduce the incidence of gastric cancer by 19% [7]. There are many transmission routes of *H. pylori*, including fecal–oral, gastric–oral, and oral–oral pathways. Age, socioeconomic status, race, and geographical region are all significant factors that influence the occurrence of infection. Moreover, the incidence rate in developing countries is higher than that in developed countries, with person-to-person and contaminated water transmission regarded as the primary modes; the infection rates among men and adults are higher than those among women and children. Unless successfully eradicated either by antimicrobial treatment or via host inflammatory and immune responses, most infections persist for life [8,9,10,11,12,13]. At present, there are 21 subgroups of *H. pylori*. Each subgroup has a different geographical distribution and pathogenicity, resulting in variations in the incidence of diseases in different regions [14]. The diagnostic methods are divided into invasive and non-invasive categories. The invasive methods include gastroscopy, biopsy, and rapid urease test, while the non-invasive methods include breath test, serological test, etc. Among them, the breath test is the most commonly used. Effective treatment of *H. pylori* infection requires multi-drug regimens; the quadruple therapy is mainly adopted, which consists of one proton pump inhibitor (PPI), one bismuth agent, and two antibiotics [15]. The treatment regimen must be taken several times a day for at least 7 days, and sometimes the treatment course is even 14 days. Patients’ compliance with such complex therapy plans can be difficult to persist. Numerous research has revealed that the current quadruple therapy has obvious side effects (such as nausea, vomiting, and allergic reaction). And the rise in antibiotic resistance to frequently used antibiotics, such as clarithromycin and metronidazole, has become a serious issue in developing countries because of the large daily dosage used, affecting the eradication rate of *H. pylori*. Furthermore, the successful eradication of *H. pylori* infection by antimicrobial treatment cannot provide persistent protection against the infection. In a number of countries, the rate of reinfection in patients who have achieved initial successful eradication is as high as 15–30% per year [16].

Perhaps most importantly, numerous people with *H. pylori* infection who develop gastric cancer typically remain asymptomatic until the cancer has progressed to the late stage, leading to difficulty to cure. The costs of offering better diagnostic testing and treatment plans to all infected people are astonishing. Among China, Japan, South Korea, and other Far East countries, as well as many Eastern European countries worldwide, getting vaccinated in populations with an increasing risk of gastric cancer could reduce the incidence of gastric cancer even if people do not have treatment to combat the *H. pylori* infection. It has been shown that a 10-year vaccination plan would significantly reduce the prevalence of *H. pylori*-related peptic ulcers and gastric cancer in the population and related morbidity and economic costs associated with these diseases in the developed countries [17]. Under the condition, vaccination against *H. pylori* infection could be administered either as a means of preventive or therapeutic treatment. In areas where *H. pylori* is endemic, preventive vaccination would be given to young children, and a therapeutic vaccine would be given to the adult population where the incidence of gastric cancer is high. Although it may be possible for the vaccine to lose its efficacy due to the time of vaccination, it could also reduce the cost of treatment, increase the effect of conventional treatment with antibiotics, and help to prevent reinfection. For these reasons, research towards a vaccine against *H. pylori* infection for use in humans has been ongoing since shortly after the isolation of *H. pylori* in 1984 [18]. We should not only optimize the management of antibiotics, but also actively develop vaccines related to *H. pylori* [19].

Since the smallpox pandemic in the 17th century promoted the development of vaccines, vaccine technology has been constantly innovated. From the initial attenuated live vaccines and inactivated vaccines, to subunit vaccines and recombinant gene vaccines, then to recombinant viral vector vaccines and nucleic acid vaccines, and up to the current nanoparticle vaccines, each vaccine has its advantages and disadvantages. Live attenuated vaccines pose risks to immunocompromised individuals, while inactivated vaccines have the potential danger of virus reactivation. The immunogenicity of subunit vaccines and recombinant gene vaccines may be insufficient [20]. Based on the limitations of traditional vaccines and increasingly complex pathogens, the research on recombinant viral vector vaccines, nucleic acid vaccines, and nano-vaccines has been promoted. At present, the recombinant viral vector vaccines that have been studied extensively can achieve efficient delivery and expression, stimulate CD8^+^ cell immunity, and since the viral vectors themselves have immune irritability, no adjuvants need to be added. Nucleic acid vaccines directly introduce DNA or mRNA encoding antigens into human cells and use host cells to translate and generate antigen proteins, triggering an immune response. Its advantages include fast R&D and production speed, high flexibility, strong immunogenicity, and high safety. Nano-vaccines can achieve precise delivery and protection, have strong immunogenicity, can display multivalent antigens, have good stability, and have dual applications of “prevention and treatment” [21,22]. The development of new vaccines brings both opportunities and challenges. The new vaccine paradigm is no longer limited to prevention but also shows broad prospects in therapeutic vaccines. Driven by new technologies, *H. pylori* vaccines have also made certain progress.

This article systematically summarizes relevant studies on the structure, pathogenicity, the immune evasion mechanism, and vaccines of *H. pylori* in recent years, and focuses on discussing the mechanism by which vaccines exert their functional effects, production principles, immunogenicity, and safety in the development of current *H. pylori* vaccines, aiming to provide new ideas for developing efficient *H. pylori* vaccines.

## 2. The Structure and Pathogenicity of *H. pylori*

*H. pylori* infection can damage the gastric mucosal barrier, adhere to and destroy gastric epithelial cells, and trigger acute inflammation dominated by neutrophils and chronic inflammation dominated by lymphocytes [23]. The pathogenic mechanism stems from the unique virulence factors of the *H. pylori* genome, which has a high level of genetic plasticity and extensive geographic variation, intricately linked to its ability to colonize in the human stomach and induce gastric disease. This highly heterogeneous bacterium is reflected in various genes associated with gastric cancer development, with a focus on cytotoxicity-associated gene (Cag) pathogenicity island (CagPAI), the vacuolating cytotoxin A (VacA), urease, flagella, the hopQ adhesin gene, sialic acid-binding adhesin (SabA), outer inflammatory protein (OipA), the blood group antigen-binding adhesin (BabA), and so on (Table 1).

The cytotoxin-associated gene A (CagA) is hidden within the pathogenic island of the cagPAI, encoding an immunodominant protein of 120–145 kDa which is translocated into gastric epithelial cells via the type IV secretion system (T4SS). After the Glu-Pro-Ile-Tyr-Ala (EPIYA) motif of CagA is phosphorylated by the host SRC/ABL kinase, CagA combines with SH2 domain-containing PTPase 2 (SHP2) to interfere with cell adhesion and polarity, activates the NF-κB pro-inflammatory pathway, induces IL-8 secretion, and co-operates with the host Wnt/β-catenin pathway to produce a persistent inflammatory microenvironment. Non-phosphorylated CagA can also bind to partitioning defective-1 (PAR1) family serine/threonine kinases, disrupt the cytoskeleton, and induce the abnormal expression of oncogenes [24,25,26]. The genetic variations of VacA are an important virulence factor of *H. pylori*, which are associated with the severity of the disease. All the strains of *H. pylori* contained the VacA gene, and the toxicity differences originated from the variations in the s/i/m/c region. VacA was produced as a 140 kDa protoxin and cleaved into a 95 kDa mature protein after secretion. VacA relies on membrane receptors such as host integrins to adhere and insert into the membrane to form channels, causing urea and nutrient leakage, destroying endosome-lysosomes, inducing vacuolation, and making host cells more sensitive to urease. The host’s susceptibility to different virulence genotypes promotes the development of infections in different pathological directions, such as gastric ulcers and gastric cancer [24,27]. The VacA can inhibit key genes of autophagy, leading to mitochondrial dysfunction and exacerbation of oxidative stress [28], and mediate the increase in DNA damage and repair defects in the host, thereby increasing the risk of carcinogenesis [29], and promote the self-spheroid transformation, which is conducive to its survival in the host [30].

In the pathogenic mechanism of *H. pylori*, biological and host factors can exert a synergistic effect through the ring of “DNA damage-repair regulation”, causing DNA double-strand breaks (DSBS) to accumulate in repetitive sequences and high transcriptional regions. Meanwhile, the CagA protein inhibits the repair signal pathway and down-regulates p53, hindering the cell cycle. The binding of host TLR2 to the bacterial urease B subunit (UreB) activates hypoxia inducible factor-1α (HIF1α), which may intensify the depletion of deoxyribonucleo-side triphosphate (dNTP). The inhibition of p53 by CagA weakens the damage response, resulting in the failure and accumulation of DSB repair and the formation of carcinogenic chromosomal aberrations (such as copy number variations in gastric cancer driver genes) [29].

*H. pylori* achieves colonization in the stomach through the movement of the spirilla and flagella, the production of ammonia by urease to neutralize gastric acid, changing the mucus environment, and inhibiting the secretion of gastric acid by the host [23,24]. The paralyzed flagellar proteins (including FlgA, FlgV, PflA, and PflB) are of vital importance for the flagellar function, facilitating bacterial motility and immune evasion [31,32]. Outer membrane vesicles (OMV) and outer membrane proteins (OMP) carry components such as VacA and urease, enter cells through the host endocytosis mechanism, trigger pro-inflammatory responses by regulating pathways such as NF-κB, and their surface adhesins can also simulate host molecules to evade immune surveillance, involved in epithelial injury and carcinogenesis [31]. Key virulence factors such as OipA, SabA, and BabA are also related to inflammation and immune evasion. Moreover, they achieve precise colonization and mucosal adhesion with the help of the host glycosylated receptors, evading immune clearance. Virulence proteins such as OipA activate signaling pathways such as the STAT1-IRF1 signal pathway in the host, induce the secretion of IL-8 and the nuclear translocation of β-catenin, and promote inflammation and cell proliferation. Virulence factors induce micronucleus formation in gastric epithelial cells, interfere with iron metabolism and glutathione homeostasis, promote abnormal cell proliferation, and stimulate the host immune system to produce autoantibodies, exacerbating immune disorders [24,33,34]. Some studies have also found that *H. pylori* pathogenicity is involved in the flagellar motility and immunological response modulation by inducing the release of inflammatory factors, which recruit immune cells and promote inflammation, contributing to the persistent infection and mucosal damage [30,35,36].

## 3. The Immune Evasion Mechanism of *H. pylori*

The CagA protein plays a crucial role in immune evasion, which can affect host cells through various mechanisms, including promoting inflammation, performing epigenetic modifications, and collaborating with the T4SS system. As a result, CagA alters gene expression, activates oncogenic signal pathways, reprograms metabolism, and ultimately significantly influences the transcriptome and proteome, leading to abnormal proliferation, apoptosis, and carcinogenesis [37]. For instance, CagA can inhibit the function of CD8^+^ T cells, render the immune protective memory response ineffective, induce senescence of gastric epithelial cells and secretion of pro-inflammatory factors, form secretory phenotype (SASP), and mediate long-term inflammation-induced immunosuppression, thereby promoting the proliferation, invasion, and metastasis of tumor cells. Even CagA-negative strains can promote bacterial colonization by recruiting immunosuppressive CD4^+^ T cells [28]. CagA also enhances programmed death ligand-1 (PD-L1) stability by up-regulating the expression of squalene epoxidase (SQLE) and inhibits T cell activation and anti-tumor immune responses [38]. Targeting the pro-apoptotic protein FADD inhibits programmed cell death and helps bacteria and cancerous cells evade clearance [39].

The difference in the duration of CagA’s entry into host cells through T4SS will lead to different disease progression. In the short term, it activates pro-inflammatory pathways such as NF-κB to trigger the Th1/Th17 immune response. Meanwhile, chronic infection leads to abnormal receptor β chains of tissue-resident memory T cells, which are replaced by immunomodulatory CD4⁺ T cells, weakening local immune surveillance [28]. In the long term, it leads to immune exhaustion and tissue damage: on the one hand, it induces cell cycle arrest and glandular structure destruction in the gastric mucosa, forming an immunosuppressive microenvironment; on the other hand, chronic inflammation of the host (such as elevated IL-1β and TNF-α) inhibits bacterial activity and promotes its escape and clearance in an inactive form. Animal models have shown that infection with cag PAI-positive strains can significantly up-regulate gastric tumor markers (CD44, KRT7) and down-regulate the tumor suppressor factor, enhancing the risk of gastric cancer through the “inflammation-escape” dynamic balance. This also provides potential therapeutic strategies for targeting the CagA-T4SS axis or regulating the immune microenvironment [37].

At the epigenetic regulatory level, virulence factors such as CagA reshape gene expression through multiple pathways: firstly, they mediate abnormal DNA methylation, leading to the silencing of tumor suppressor genes related to the p53 pathway and the activation of oncogenic pathways such as the Wnt signal pathway; secondly, they regulate the miRNA network. For example, the intact cag PAI strain induces overexpression of pro-cancer miR-21 and down-regulation of tumor suppressor miR-34a through CagA, promoting abnormal cell signaling pathways and uncontrolled proliferation, continuously activating the NF-κB pathway, and promoting tumor migration, invasion, and microenvironment remodeling [28,39].

Apart from CagA, Toll-like receptors (TLRs) also play an important role in *H. pylori*’s immune evasion. Lipopolysaccharide (LPS) and flagellin of many bacterial pathogens are well-described ligands that are detected by TLRs. The LPS of Gram-negative bacteria is prominently displayed on the cell surface, serving as a protective barrier against external threats. It is composed of three distinct regions: an acylated lipid A, an oligosaccharide core, and outer polysaccharides. The disaccharide backbone of lipid A is linked to phosphorylated sugars, which confer a negative surface charge. The core oligosaccharide is synthesized at the inner periplasmic membrane and subsequently transported to the outer surface, where it is attached to lipid A. In *H. pylori*, lipid A is synthesized at the periplasmic membrane as hexa-acylated lipid A. It is then translocated across the inner membrane, modified, conjugated with the core oligosaccharide, and ultimately transported to the outer membrane via the periplasmic space. Upon passage through the periplasmic space, acyl chains of the newly assembled LPS are enzymatically deacylated. Analogous to LPS, the flagellin of *H. pylori* possesses specific attributes that enable it to evade detection by TLR5. A chimeric construct of *Salmonella* FliC, incorporating the N-terminal D0-D1 domain of *H. pylori* FlaA, was completely ineffective in stimulating TLR5 signaling. The motif responsible for this evasion is attributed to amino acid residues 89–96 of the N-terminal D1 domain. Introduction of these specific amino acids into FliC resulted in the complete abolition of TLR5 agonist activity. This sophisticated study clearly illustrated the limited recognition of *H. pylori* flagellin by TLR5 [40]. TLR2 can bind to bacterial components (such as LPS, lipoproteins, peptidoglycan PGs), as well as the neutrophil-activating protein of *H. pylori*, and form hetero-dimer receptor complexes with TLR1 or TLR6, thereby activating TLR2. This process recruits myeloid differentiation primary response protein 88 (MyD88), and then successively activates members of the IL-1 receptor-associated kinase family (IRAK), ultimately leading to the activation of tumor necrosis factor receptor-associated factor 6 (TRAF6). TRAF6 activates transforming growth factor—β -activated protein kinase 1 (TAK1). On the one hand, TAK1 phosphorylates and activates the classic IκB kinase (IKK) complex, enabling the activation of NF-κB. On the other hand, mitogen-activated protein kinases (MAPKs) are activated, indirectly affecting the activity of NF-κB through a series of cascade reactions. *H. pylori* may evade immune clearance by interfering with TLR2 signals, such as secreting inhibitory proteins. TLR4 needs to form a heterodimer (TLR4/MD2 complex) with the MD2 protein to recognize LPS, trigger the activation of the NF-κB signaling pathway, and subsequently activate the IL-8 pathway to promote the release of pro-inflammatory cytokines. *H. pylori* can modify the lipid A core of its LPS to achieve immune escape. TLR7/8 also recognizes the RNA of *H. pylori* [24,33,40]. TLR9 is an endosomal receptor that can trigger an immune response by recognizing the glycocalyx of Hp. Pylori. *H. pylori* DNA can activate the microbial DNA sensor TLR9 in vitro and that TLR9 suppresses *H. pylori*-induced injury in vivo [24,41,42]. During the acute infection period, TLR6 rapidly activates the inflammatory pathways. However, during the chronic infection period, the sensitivity of TLR6 to *H. pylori* components gradually decreases, resulting in immune tolerance [43].

In addition to transmembrane receptors on the cell surface and in endosomal compartments, soluble cytosolic receptors, RIG-like receptors detect the presence of intracellular pathogen-associated molecular patterns. The retinoic acid inducible gene-I (RIG-I) induces type I interferon (IFN) in response to different RNA viruses. Recently, it has been shown that 5′-triphosphate RNA, which is generated during infection with most RNA viruses, interacts with RIG-I to induce the IFN response. *H. pylori* RNA can act as a specific RIG-I ligand and may thereby contribute to the induction of MyD88-independent type I IFN expression. *H. pylori* disrupts the host’s defense through multiple pathways, such as inhibiting the stimulator of interferon genes (STING)/RIG-I signal pathway to reduce interferon production; weakening the functions of neutrophils/macrophages; inhibiting T cell proliferation; and inducing an imbalance in Treg, Th1, and Th17 immune responses [44]. *H. pylori* down-regulates STING and IRF3 activation but induces autophagy in human gastric organoids. Trim30a, a known Sting suppressor, is up-regulated by *H. pylori* in vivo in a Sting-dependent manner [45]. *H. pylori* can lead to the defect of neutrophil uropod contraction, hinder the migration of neutrophils to the infection site, and finally affect the function of neutrophils. *H. pylori* can also produce reactive oxygen species (such as H_2_O_2_), causing mitochondrial membrane depolarization, activating apoptotic executive proteins, and interfering with cellular metabolic homeostasis through polyamine-dependent pathways, thereby promoting macrophage apoptosis. The phospholipase A (PldA) secreted by *H. pylori* promotes its survival by destroying the membrane of macrophages [46]. The protease system achieves evasion of immune surveillance by degrading NKG2D ligands [47]. The above-mentioned multiple factors jointly construct an immunosuppressive microenvironment conducive to its persistent infection.

The effective immune response against *H. pylori* requires multiple effects (Table 2): firstly, it directly intervenes in the pathogen by blocking bacterial colonization (such as inhibiting adhesion binding) and neutralizing virulence factors (such as targeting the CagA-T4SS system); secondly, it activates the host immune response (such as driving the Th1/Th17 response and enhancing the function of CD8⁺ T cells) and reverses the immune escape mechanism (restore immune surveillance by regulating epigenetic modifications, metabolic reprogramming, etc.). Ultimately, these strategies were integrated into the vaccine design to construct a vaccine that can effectively prevent and treat *H. pylori*. In the future, we could pay more in-depth attention to the immune evasion mechanisms to discover new therapeutic targets and effectively prevent and treat related diseases.

## 4. Vaccines Related to *H. pylori*

### 4.1. Vaccine Technologies of H. pylori

At present, there are no approved and marketed products for *H. pylori*. However, significant progress has been made in related research, which includes the following four types of vaccines: whole-cell vaccine, subunit vaccine, live vector vaccine, and DNA vaccine (Table 3).

The whole-cell antigen of *H. pylori* can be obtained by ultrasonic disruption or formalin inactivation of the bacteria. The inactivated whole bacterial vaccine designed by Kotloff et al. underwent Phase I clinical trials and was proven to effectively stimulate the mucosal and systemic immune response of the human body to *H. pylori* antigen [48]. Another study utilized cholera toxin as an adjuvant to develop an inactivated whole-cell vaccine against *H. pylori*, which was confirmed to induce immune responses in a mouse model [49]. Although whole-cell vaccines can elicit efficient local mucosal immune responses, they have relatively large side effects. Due to their complex antigenic components, they are prone to causing immune diseases, thus limiting their clinical application [50]. Researchers are committed to developing safer alternatives.

Subunit vaccines utilize recombinant protein antigens and possess excellent immune protection mechanisms because the vaccines have a strong targeting ability and high safety [50]. Studies have shown that the oral subunit vaccine of *H. pylori* can trigger Th1/Th17 responses, stimulate the production of secretory IgA, and significantly enhance the ability to clear pathogens [51]. In the production of subunit vaccines, host cells act as bioreactors and can efficiently express recombinant protein antigens. For instance, *Escherichia coli* can be used to express specific *H. pylori* antigen proteins. By optimizing culture conditions and regulating gene expression, the yield and quality of antigens can be enhanced. Using *H. pylori* antigens, including CagA, VacA, UreB, and neutrophil-activating protein (NapA), as targets and cholera toxin subunit B (CTB) as adjuvant, the multi-epitope subunit vaccine was constructed, which alleviated the colonization of *H. pylori*, the degree of gastric inflammation, and atrophy. Sarabi et al. predicted through C-ImmSim simulation that it could induce strong humoral/cellular immunity. After optimizing the protein sequence with JCat, it was cloned into pET28b (+) (7.053 kb). However, animal experiments were not continued for verification [52]. A study in 2020 found that after BALB/c mice were orally administered a multiepitope vaccine containing NAP, UreA and UreB, Th1/Th17 polarization (elevated IFN-γ/IL-17), antigen-specific lymphocyte proliferation, and antibody response were detected in their gastric, splenic, and mesenteric lymph nodes, and gastric inflammation was also significantly alleviated [53]. There are also multi-epitope subunit vaccines based on B cells and T cells, which have been verified to exert their effects by binding to Toll receptors through molecular docking [54,55,56]. A study constructed a spherical multi-epitope nanoparticle vaccine and verified its potential for inducing Th1/Th17 responses and antibody generation with online tools [32]. A multivalent vaccine, LHUC fusion peptide, developed in 2021, has been proven to significantly reduce the *H. pylori* load in the stomach, neutralize urease/catalase activity, alleviate inflammation, and have both preventive and therapeutic effects in both BALB/c and C57BL/6 mouse models [57]. Peptide vaccines targeting CagA, VacA, and SabA were immunized four times in BALB/c mice. Serum IgG/IgA and IL-4/IL-17 were detected to increase significantly, and the level of gastric inflammation was much lower than that of the control group [58]. The α-1, 6-glucano-carrier protein-coupled vaccine synthesized using α-1, 6-glucosyltransferase can produce highly specific IgG after immunizing mice/rabbits and can accurately identify clinical isolates (including drug-resistant bacteria). This technology provides a new method for designing synthetic carbohydrate vaccines against *H. pylori* [59]. Although the subunit vaccines induce an immune response in the host, their protective effect against *H. pylori* infection is relatively low. Which antigen and the sub-structure of *H. pyloti* to form a multivalent vaccine that can achieve the optimal immune efficiency still require in-depth research.

Live vector vaccines carry the antigen genes of *H. pylori* through attenuated bacterial or viral vectors, simulating natural infection to activate the immune response. By using bacteria as host cells, the *H. pylori* antigen gene can be introduced into the bacterial genome through genetic engineering technology, enabling it to express the antigen during the bacterial growth process. Several recombinant vaccines using *Lactobacillus* as vectors have shown effects in activating IgG/sIgA, inducing Th1/Th2 balance and multi-cytokine secretion, and have all been demonstrated in animal models to reduce bacterial load and alleviate gastric inflammation. However, only one LL-plSAM-WAE vaccine studied in 2022 found that the activation of the immune system led to an increase in the inflammatory response in the stomach [60,61,62,63,64]. Multi-antigenic epitope vaccines using *Salmonella* as vectors have also been proven to have a good effect in activating the Th1/Th2/Th17 response. The *Salmonella* vector vaccine constructed by Ghasemi et al. has also been shown to have a certain effect in reducing gastric inflammation caused by other bacteria [65,66]. The influenza virus vector SSN-NAPA vaccine can activate NAPA-specific Th1/Th17 cells and has a strong effect in both the prevention and treatment of *H. pylori* infection [67]. Oral vaccines using *Bacillus subtilis* and yeast as vectors can also induce mucosal sIgA and systemic IgG. Moreover, the thermal stability and progenicity of *Bacillus subtilis* spores support their potential as oral vaccines [68,69]. Attenuated Shigella vector vaccines have been studied mainly for antibody-mediated sterilization, cellular immune regulation of inflammatory damage, and also have a certain role in preventing *H. pylori* infection [70]. Due to the high technical difficulty and potential biological safety risks, no breakthrough progress has been made in live vector vaccines of *H. pylori*.

The DNA vaccine, through the nucleic acid route, can induce the body to generate a comprehensive immune response [50]. In the production of DNA vaccines, Escherichia coli is often used as the host cell to amplify plasmid DNA. By introducing plasmids containing the target antigen gene into Escherichia coli, under suitable culture conditions, Escherichia coli multiplies in large quantities and replicates the plasmids. Subsequently, high-purity DNA vaccines are obtained through steps such as extraction and purification. A study constructed a multi-epitope DNA vaccine using reverse vaccinology, with CPG/C274 as an adjuvant, which significantly enhanced the level of IgG and could induce Th1-type immunity and long-term memory. This study achieved, for the first time, the complete blocking of *H. pylori* colonization by a multi-epitope vaccine [71]. The Alginate/pCI-neo-UreH vaccine, constructed based on the UreH protein, alginate vector can improve DNA delivery efficiency, activate Th1/Th17 response and mucosal sIgA, and the process of mucosal IgA response does not rely on the traditional Th17/IL-17R signaling pathway. There may be other regulatory pathways (such as direct activation of B cells by Th1 cells and dendritic cells, or the direct effect of the local mucosal microenvironment, etc.) [72]. There are also DNA vaccines such as lipoplex-A vaccine, pIRES2-DsRed-Express-ureF DNA vaccine, and pBud-CE4.1-flaA vaccine, all of which induce Th2-dominated mixed responses [73,74,75]. A chitosan nanoparticle (pcDNA3.1(+)-cagW-CS-NPs) vaccine studied in 2020 induced a Th1/Th2 mixed response. Moreover, chitosan nanoparticles could protect DNA from degradation and enhance antigen expression. In vivo experiments showed that it could significantly reduce the bacterial load in the stomach, prevent infection and death, and had no tissue damage. It further proved the potential of the *H. pylori* DNA vaccine [76]. Antigen selection is of vital importance for the immune response. Research has found that CagA, CagW, CagL, VacA, urease antigen, outer membrane protein, etc., are excellent candidate antigens [77,78]. Furthermore, the chemically fully-synthesized 3-D glycerol-D-mannopyranose antigen also demonstrates potential as a vaccine candidate for preventing and treating *H. pylori* [79]. Although DNA vaccines have significant advantages over traditional vaccines, their clinical application still faces two major challenges: genomic integration risk and insufficient immune efficacy. Studies have shown that *Helicobacter pylori* DNA may induce abnormal transformation of the host genome. If it invades reproductive cells, it will pose a risk of genetic cascade. Meanwhile, the immune effect shows a clear animal size dependence: experiments on mice demonstrate a highly efficient antibody response, but the immunogenicity is significantly reduced in large mammals, which brings a major technical bottleneck for human application [80].

Adjuvants also play an important role in the immune response to vaccines. Recombinant or mutant forms of heat-labile cholera toxin (CT) or heat-stable enterotoxin (LT), cytokines (such as IL-18, IL-17A, IL-22, etc.), chitosan, etc., can all be used as effective adjuvants [50,81,82]. In particular, chitosan nanoparticles have been confirmed by numerous studies to significantly enhance the immune response [83,84]. Other adjuvants such as granulocyte-macrophage colony-stimulating factor (GM-CSF), lipids, polyinosinic-polycytidylic acid (poly I: C), and exosomal vesicles can also effectively enhance the immune response induced by vaccines [85,86,87,88]. These data proved that vaccines produced with adjuvant as a new candidate antigen have significant value in controlling *H. pylori* infection. At present, there are relatively few studies on the *H. pylori* adjuvant vaccine. The mechanism of adjuvants in diseases caused by bacteria has not been fully clarified, and there are still many problems to be solved for the successful development of the *H. pylori* adjuvants vaccine.

The mucosal and systemic *Helicobacter pylori* vaccines of experimental mice can significantly reduce the bacterial load and even achieve sterilized immunity. Clinical trials of oral vaccines that combine *Helicobacter pylori* protein with bacterial exotoxin adjuvants or use attenuated live bacterial vectors expressing this protein can induce adaptive immunity, but they cannot continuously reduce the bacterial burden. Clinical and mouse studies have shown that regardless of whether *Helicobacter pylori* is cleared spontaneously or by vaccination, the host will develop cellular immunity. New vaccine formulations containing multiple antigens and optimizing cellular immunity may enhance efficacy. However, the industrial sponsors who once strongly supported numerous animal and human research no longer do so [80].

### 4.2. Production Principles of H. pylori Vaccine

#### 4.2.1. Mucosal Vaccine Design and Molecular Adjuvant Technology

Mucosal immunity takes priority: The gastric mucosa is the main site for *H. pylori* colonization. Mucosal immunity can generate a specific immune response locally, which is compatible with the natural infection route of *H. pylori* and can significantly reduce the colonization of *H. pylori*. Moreover, mucosal immunity can also induce a systemic immune response and reduce the side effects caused by traditional injection routes. Currently, most vaccines are designed for mucosal immunity. The vaccine studied by Azami et al. achieved the complete blocking of *H. pylori* colonization for the first time [71]. The vaccine studied by Katsande, Zhang et al. not only exerted mucosal immunity but also induced a systemic immune response [68,70].

Molecular adjuvants: Adjuvants play a crucial role in the production of vaccines. They can exert their effects by enhancing immunogenicity and reducing side effects. For instance, the adjuvant CPG/C274 used in the vaccine of Azami et al. significantly enhanced the immune response [71].

#### 4.2.2. Immunogenicity, Safety and Stability

Immunogenicity is the basis for vaccines to exert protective effects. The ideal vaccine should be capable of inducing specific immune responses and generating lasting immunity. Many studies have emphasized the importance of immunogenicity when selecting antigens in the design of vaccines. Moreover, the vaccines we designed should not cause excessive antibody reaction risks, pathogenicity, or allergenicity [61]. Additionally, during the vaccine design process, we should fully consider the health risks of special populations (such as the elderly, pregnant women, children, people with low immunity, etc.). In addition, in the existing vaccine research, it has also been reflected that in the process of designing vaccines, the stability of vaccines should be considered, including their physical stability, chemical stability, and thermal stability, to ensure that vaccines do not deteriorate during storage and transportation [32,67,71].

#### 4.2.3. Cost and Production Feasibility Control

Most of the various vaccines introduced above are in the preclinical research stage. If they are to be mass-produced, cost and production feasibility analysis are very important. We need to overcome key technologies such as genetic engineering, antigen purification, and nano-encapsulation. To increase public acceptance, we also need to control costs to keep the selling price lower than that of traditional antibiotic treatment.

### 4.3. The Production Plan of H. pylori Vaccine

At present, there are similar production schemes for subunit vaccines. Firstly, suitable targets and adjuvants are selected. Then, epitope prediction is carried out through some online tools. Different connectors (such as GPGPG, AAY, etc.) are used to connect and generate multi-epitope vaccines. Then, codon optimization is performed through various online programs such as JCat. The optimized sequences are cloned into specific vectors. Likewise, some online servers were used for property analysis and simulation. Finally, animal experiments were conducted (mostly BALB/c mice and C57BL/6 mice were selected as models) to detect the type and effectiveness of the immune response induced by the vaccine [54,55].

In the production of live vector vaccines, our first step is to select appropriate vectors and antigens. Through molecular cloning technology, the antigen genes are cloned into the plasmids of the vectors to construct recombinant strains. Then, the expression of antigen proteins is verified by methods such as Western blot, immunofluorescence, and ELISA. Vector vaccines capable of expressing current antigens can be prepared through means such as physical adsorption and gene fusion. Subsequently, the characteristics of the vaccines (including morphology and size, antigen carrying verification, targeting verification, etc.) are analyzed. Then, the immune response (antibody level of the mass, cytokines, etc.) and anti-infection effect (bacterial load of the mass, urease activity, histopathological analysis, etc.) were detected through animal experiments [60,65].

The production plan of DNA vaccines roughly follows the following process. Firstly, the authenticity of the protein is verified using the database. Potential vaccine targets are screened by analyzing subcellular localization (prioritizing surface proteins) and confirming sequence specificity (avoiding host cross-reactions), and then their reliability, antigenicity, stability, etc., are verified. Subsequently, through computer-aided codon optimization, recombinant plasmids were constructed using molecular cloning technology. The constructed recombinant plasmids were verified by enzyme digestion, sequencing, PCR, etc., to prepare DNA vaccines or nanocell treatment (optional). The expression of the vaccine was verified by methods such as real-time-polymerase chain reaction (RT-PCR), Western blot, and immunofluorescence. Finally, the relevant indicators of humoral immunity (such as detecting the concentration of immunoglobulin and antibody subtypes in serum by ELISA), cellular immunity (such as detecting cytokines and Th cell subtypes by PCR technology and flow cytometry), and anti-infection effect were detected through animal experiments (such as PCR, Gram staining, rapid urease assay, detection of bacterial load, pathological tissue analysis, etc.) [71,72,76].

Although the research and development of *H. pylori* vaccines has achieved certain progress, in the future, we need to optimize the safety, immunogenicity, and antigen selection of the vaccines, and explore new adjuvants, in order to achieve more effective prevention and treatment strategies and promote the rapid market launch of *H. pylori* vaccines.

## 5. Summary and Outlook

The effectiveness of antibiotic-based therapies for *H. pylori* infection presents significant challenges. Current treatment regimens are constrained by the rapid degradation of antibiotics in acidic environments, their limited ability to penetrate the gastric mucus layer and biofilm, and their inadequate uptake by gastric epithelial cells to eliminate intracellular *H. pylori*. Furthermore, existing triple or quadruple therapy protocols necessitate adherence to complex medication schedules over prolonged durations. Inadequate treatment may fail to eradicate *H. pylori*, whereas excessively prolonged therapy can lead to adverse effects such as diarrhea, nausea, taste disturbances, rashes, and abdominal pain, thereby affecting patient adherence. Additionally, prolonged and inconsistent drug use may contribute to increased bacterial resistance, complicating the eradication of *H. pylori*. The administration of PPIs and antibiotics can also disrupt the gut microbiota, heightening susceptibility to intestinal infections and thereby reducing the success rate of *H. pylori* eradication. This situation imposes substantial physical, psychological, and economic burdens on patients. While medical devices present innovative strategies for the eradication of *H. pylori*, they are accompanied by a distinct set of challenges and burdens. The enforcement of new medical device regulations poses significant challenges for businesses, primarily due to the increased resource expenditure and costs linked to technical documentation and quality management systems. This additional administrative burden has the potential to adversely affect the accessibility and affordability of these devices for patients. Furthermore, the utilization of medical devices may introduce new challenges concerning patient compliance and acceptance. For example, the requirement for specialized procedures or the regular use of medical devices can impose a substantial burden on patients, particularly if it necessitates frequent hospital visits or if the devices are complex to operate. Given these challenges, there is a critical need to develop an integrative medical approach for the eradication of *H. pylori*.

A study in Japan indicates that screening and eradication of *H. pylori* during the school-age period can reduce the risks of gastritis, gastric ulcer, and gastric cancer. It is recommended that such measures be incorporated into the routine physical examinations in schools [89]. Although the initial screening and treatment require certain economic costs, in the long run, eradicating the infection can significantly reduce the incidence and mortality of gastric cancer, bring health benefits, and save medical resources and social costs [90,91,92]. Vaccination against *H. pylori* can reduce the occurrence of related diseases, bacterial colonization, and the antibiotic resistance rate. Therefore, it is very necessary to develop the vaccine [81]. At present, the research and development of *H. pylori* vaccines is very active internationally, but there are no related marketed vaccines yet [93]. Most of the above-mentioned vaccine types are in the preclinical research stage. Only the novel *H. pylori* vaccine designed by Professor Zou Quanming’s laboratory, which fuses the urease B subunit with the thermally unstable enterotoxin B subunit, has completed Phase 3 clinical trials, and this vaccine has been proven to be effective, safe, and immunogenic [94]. To develop a qualified *H. pylori* vaccine, many features that play critical roles in influencing the success of *H. pylori* vaccines need to be considered. Choosing the appropriate antigen is of vital importance, and AI algorithms can help in designing antigens with strong immunogenicity [51]. At the same time, issues such as dosage, vaccination route, safety, formulation strategies, immunity persistence, the capacity to stimulate specific protective immune responses, prioritization, and the production cost need to be taken into consideration [95]. Given the differences in subgroups infected by different races and antibiotic resistance among them, vaccine design should fully take into account racial factors [96]. With the application of nanotechnology in the medical field, its potential in vaccines and immunotherapy has been confirmed [97,98,99]. In the future, nanotechnology may be utilized to optimize the research and development of *H. pylori* vaccines to achieve better results.

This review has concentrated on the pathogenic mechanism of *H. pylori* and the mechanism of evading the immune attack of the host, and the *H. pylori*vaccine shows significant promise in reducing the global burden of infection. However, there are still challenges related to safety, efficacy, and availability of the HP vaccine. Future research should aim to elucidate the specific role of the *H. pylori* vaccine in the immune response to treatment, investigate more effective prevention and treatment strategies for *H. pylori* infection, and explore the specific mechanisms by which *H. pylori* infection influences the prognosis of immunotherapy. This review lays the groundwork for new strategies and directions for the development of responsive vaccines that are more efficacious at eradicating *H. pylori* and protecting the intestinal microbiota.

## 6. Materials and Methods

This article conducted a search in Pubmed using different combinations of search terms such as “vaccine” (and its derivatives), “*Helicobacter pylori*”, “*Helicobacter pylori* vaccine”, “whole bacterial vaccine”, “subunit vaccine”, “DNA vaccine”, “live vector vaccine”, “*Helicobacter pylori* immune escape”, and “*Helicobacter pylori structure*” (and its derivatives) from 1984 to 2025. For articles published in the last 41 years, full-text articles published in English were selected. Refer to the reference lists of recent reviews and research reports to find more relevant articles.

## Figures and Tables

**Table 1:** The pathogenic mechanism of *H. pylori*.

Virulence Factor/Gene	Structural/Genetic Characteristics	Function/Pathogenic Mechanism	References
CagA	Located on the pathogenic island of cagPAI, it encodes an immune dominant protein of 120-145 kDa and enters host cells via T4SS	Activate the secretion of IL-8 and trigger a carcinogenic cascade reaction (disrupting cell polarity and causing DNA damage). After phosphorylation, it binds to SHP2, interferes with cell adhesion, and activates the NF-κB inflammatory pathway. Non-phosphorylated CagA binds to PAR1 to induce abnormal expression of oncogenes.	[24,25,26]
VacA	Genetic variation (s/i/m/c region) affects toxicity; Secreted as 140 kDa prototoxin and cleaved into 95 kDa mature protein	Form membrane channels, destroy organelles, and induce apoptosis. Inhibiting autophagy genes leads to mitochondrial dysfunction and oxidative stress. Mediating DNA damage repair deficiencies increases the risk of canceration. Promote self-sphere transformation and enhance survival ability	[24,27,28,29,30]
Urease	Urea is decomposed to produce ammonia, which neutralizes gastric acid	Alter the mucus environment and inhibit gastric acid secretion. The binding to the host TLR2 activates HIF1α, which intensifies the depletion of dNTP.	[23,24,27]
Flagella-associated proteins (FlgA, FlgV, etc.)	Proteins related to the morphology and flagellar motility of Spirilla	Promote bacterial movement and immune escape.	[23,24,31,32]
OMV and OMP	It carries components such as VacA and urease	It enters the host cells through endocytosis and activates the pro-inflammatory response of NF-κB. Surface adhesins mimic host molecules, evade immune surveillance, and are involved in epithelial damage and carcinogenesis.	[31]
OipA	Outer membrane inflammatory protein	Activate the STAT1-IRF1 signaling pathway, induce IL-8 secretion and β-catenin nuclear translocation, and promote inflammation and cell proliferation	[20,31,32]
SabA and BabA	Sialic acid-binding adhesive and blood group antigen-binding adhesive	It binds to the host glycosylated receptor to achieve precise colonization and mucosal adhesion. Evading immune clearance aggravates inflammation and immune disorders	[24,33,34]
Synergistic pathogenic mechanism	Through the “DNA damage—repair regulation” loop	CagA inhibits the repair signaling pathway, down-regulates p53, and hinders the cell cycle. Accumulation of DNA double-strand breaks (DSBs) leads to carcinogenic chromosomal aberrations (such as copy number variations in driver genes).	[29]

**Table 2:** The immune escape mechanism of *H. pylori* and related therapeutic strategies.

The Immune Escape Mechanism and Function of CagA Protein			
Virulence Factor/Mechanism	Mode of Action	Function/Pathogenic Mechanism	References
CagA protein	It enters the host cells through the T4SS system and affects gene expression and signaling pathways.	Immunosuppression: inhibits the function of CD8⁺ T cells and induces immune memory failure; promotes the recruitment of regulatory CD4⁺ T cells (even for CagA negative strains). Pro-inflammatory and carcinogenic: activates inflammatory pathways such as NF-κB, induces cellular senescence and the secretion of pro-inflammatory factors (SASP), and forms a long-term immunosuppressive microenvironment. Immune checkpoint regulation: up-regulation of SQLE enhances the stability of PD-L1 and inhibits the anti-tumor response of T cells. Anti-apoptosis: targeting FADD to inhibit programmed cell death helps bacteria and cancer cells evade immune clearance.	[28,37,38,39]
The difference in the action time of CagA	Short-term and long-term infection dynamics affect the immune response.	Short-term: activate the Th1/Th17 immune response and trigger acute inflammation. Long-term: it leads to the exhaustion of T cells, the destruction of the glandular structure of the gastric mucosa, and the formation of an immunosuppressive microenvironment. Promotes the risk of gastric cancer through the dynamic balance of “inflammation-escape” (up-regulate CD44/KRT7 and down-regulate tumor suppressor factors)	[28,37]
Epigenetic regulation	Remodeling gene expression through DNA methylation and miRNA networks	DNA methylation: silencing p53-related tumor suppressor genes and activating oncogenic pathways such as WNT. miRNA regulation: induce overexpression of pro-cancer miR-21, inhibit tumor suppressor miR-34a, and continuously activate NF-κB to promote tumor migration and microenvironment remodeling.	[28,39]
The immune escape mechanism of TLRs			
TLR subtype	Identify the target	*H. pylori*’s escape strategy	References
TLR2	LPS, lipoprotein, PGs	It forms a heterodimer with TLR1/6 and activates the MyD88-IRAK-TRAF6-TAK1-NF-κB pathway. Secreting inhibitory proteins interferes with TLR2 signal transduction.	[24,33,40]
TLR4	LPS (needs to form a complex with MD2)	Modify the lipid A core of LPS to evade immune recognition.	[24,33,40]
TLR5	Bacterial flagellin	Down-regulate flagellar expression or alter flagellar structure.	[40]
TLR6	Bacterial components (activation of inflammation in the acute phase)	Reduce TLR6 sensitivity and induce immune tolerance in chronic infections.	[43]
TLR7/8	Bacterial RNA	Modify the RNA structure to evade immune recognition.	[24,33,40]
TLR9	glycocalyx	Utilize the anti-inflammatory mechanism of TLR9 to maintain chronic infections.	[24,41,42]
Other immune escape mechanisms			
Mechanism	Mode of action	Function/Pathogenic mechanism	References
Interfere with innate immune signals	Inhibit the STING/RIG-I pathway	Reduce interferon production and weaken antiviral immunity	[44,45]
Weaken the function of immune cells	Inhibit the migration of neutrophils and induce the apoptosis of macrophages	Prevent neutrophils from migrating to the site of infection. Macrophage cell membranes are destroyed by H_2_O_2_ and PldA to activate apoptotic executive proteins	[46]
Protease system	Degrade the NKG2D ligand	Evading immune surveillance (such as the killing function of NK cells)	[47]
Imbalance of T-cell immunity	Inhibit the proliferation of T cells and induce the imbalance of Treg/Th1/Th17	Form an immunosuppressive microenvironment	[44]
Immunotherapy strategy			
Strategy direction	Specific method	Objective	
Direct intervention of pathogens	Block bacterial colonization (inhibit adhesion). Neutralizing virulence factor (targeting the CagA-T4SS system).	Reduce the infection load and inhibit pathogenicity.	
Activate host immunity	Enhance the Th1/Th17 response and CD8⁺ T cell function. Reverse epigenetic modification and metabolic reprogramming.	Restore immune surveillance and eliminate infections.	
Vaccine design	Integrate multi-target strategies (such as CagA, VacA, adhesins, etc.).	Prevent and treat *H. pylori* infection to reduce the risk of gastric cancer	

**Table 3:** *H. pylori* vaccines.

Vaccine Type	Advantages	Limitations	Experimental Verification and Effect	References
Whole-cell vaccines	Stimulate mucosal and systemic immune responses	The side effects are relatively large.	- Oral inactivated vaccines successfully induced immune responses in volunteers. - The whole-cell vaccine of cholera toxin adjuvant is effective in mouse models.	[48,49,50]
Subunit vaccines	Strong targeting and high safety; it can induce the Th1/Th17 response and sIgA secretion.	Adjuvants are needed to enhance immunogenicity.	- Multi-epitope vaccines (CagA/VacA/BabA) can reduce the risk of immune escape. - The NAP/UreA/UreB vaccine significantly alleviates gastritis and induces Th1/Th17 polarization.	[32,50,51,52,53,54,55,56,57,58,59]
Live vector vaccines	- Simulate natural infection and activate comprehensive immunity. - Some carriers (such as Bacillus) have good thermal stability	The technical difficulty is high, with potential biosafety risks, and it may enhance local inflammation.	- Lactic acid bacteria vector vaccines activate IgG/sIgA and reduce bacterial load. - Influenza virus vector vaccine (SSN-NAPA) has both preventive and therapeutic effects.	[60,61,62,63,64,65,66,67,68,69,70]
DNA vaccines	Induce comprehensive immunity (Th1/Th17/Th2).	- Some vaccines mainly have Th2 reactions. - The delivery efficiency needs to be optimized.	- A multi-epitope DNA vaccine developed in 2024 achieved complete blocking colonization for the first time. - The Alginate/pCI-neo-UreH vaccine activates mucosal sIgA in the non-traditional Th17 pathway.	[50,71,72,73,74,75,76,77,78,79,80]
